# Evaluation of the water-equivalent characteristics of the SP34 plastic phantom for film dosimetry in a clinical linear accelerator

**DOI:** 10.1371/journal.pone.0293191

**Published:** 2023-10-23

**Authors:** Kyo-Tae Kim, Yona Choi, Gyu-Seok Cho, Won-Il Jang, Kwang-Mo Yang, Soon-Sung Lee, Jungbae Bahng

**Affiliations:** 1 Neuroscience Research Institute, Seoul National University Medical Research Center, Seoul, Korea; 2 Research and Development team, Radexel Inc., Seoul, Korea; 3 Department of Accelerator Science, Korea University Sejong Campus, Sejong, Korea; 4 Research Team of Radiological Physics & Engineering, Korea Institute of Radiological & Medical Sciences, Seoul, Korea; 5 Department of Radiation Oncology, Korea Institute of Radiological & Medical Sciences, Seoul, Korea; 6 Department of Radiation Oncology, Kangwon National University hospital, Chun-cheon, Korea; University of Malaya, MALAYSIA

## Abstract

In this study, some confusing points about electron film dosimetry using white polystyrene suggested by international protocols were verified using a clinical linear accelerator (LINAC). According to international protocol recommendations, ionometric measurements and film dosimetry were performed on an SP34 slab phantom at various electron energies. Scaling factor analysis using ionometric measurements yielded a depth scaling factor of 0.923 and a fluence scaling factor of 1.019 at an electron beam energy of <10 MeV (i.e., R_50_ < 4.0 g/cm^2^). It was confirmed that the water-equivalent characteristics were similar because they have values similar to white polystyrene (i.e., depth scaling factor of 0.922 and fluence scaling factor of 1.019) presented in international protocols. Furthermore, percentage depth dose (PDD) curve analysis using film dosimetry showed that when the density thickness of the SP34 slab phantom was assumed to be water-equivalent, it was found to be most similar to the PDD curve measured using an ionization chamber in water as a reference medium. Therefore, we proved that the international protocol recommendation that no correction for measured depth dose is required means that no scaling factor correction for the plastic phantom is necessary. This study confirmed two confusing points that could occur while determining beam characteristics using electron film dosimetry, and it is expected to be used as basic data for future research on clinical LINACs.

## Introduction

In the field of radiation oncology, interest in treatment techniques using ultra-high-dose rate (UHDR) beams has recently increased. This is a high-dose treatment that is approximately 100 times more powerful than conventional radiation therapy (Conv-RT) (≤0.4 Gy/s); the current FLASH-RT technology can exceed 100 Gy/s for electrons and 40 Gy/s for protons [1]. This is expected to be more effective than temporal interfraction, which has been widely used in the past. Various preclinical studies on the possibility of increasing the efficacy of radiation therapy by maintaining the tumor control probability in FLASH-RT and reducing the probability of normal tissue complications through the sparing effect of normal tissue have been conducted [2–6]. Based on these advantages, various research groups have invested significant time and resources in FLASH-RT implementation, and the world’s first use of high-energy electron beams for patient treatment was reported in 2019; to extract electron beam energy in the range of 4–20 MeV for clinical purposes [2, 5, 7–13].

To ensure traceability, high-energy electron beams should be measured by placing an ionization chamber in a water phantom under the reference conditions recommended by international protocols [14, 15]. However, in the case of FLASH-RT-specific equipment, performing dosimetry in a water phantom faces some issues due to irradiation surface and source-to-surface dose (SSD) limitations. Using an ionization chamber as a reference dosimeter is difficult because the dose rate correction and saturation phenomena appear due to recombination and polarity effects in the UHDR beam area [9]. Therefore, film dosimetry in FLASH-RT research is performed by placing radiochromic films (RCFs) in a water-equivalent plastic phantom [9–13]. International protocols recommend that when using white polystyrene (also known as high-impact polystyrene), no correction for the measured depth dose is required [16]. However, these brief recommendations cause some confusion while determining beam characteristics through film dosimetry in electron FLASH-RT studies: (a) the white polystyrene (ρ = 1.06 g/cm^3^) material presented in international protocols differs from the white polystyrene material widely used in the field of radiation therapy and (b) when performing film dosimetry using white polystyrene, the recommendation that correction for the measured depth dose is not required is ambiguous, particularly exactly which correction factor is not required. Therefore, we attempted to clearly present and verify some confusing points regarding performing film dosimetry with a water-equivalent phantom in electron beams.

In this study, we experimentally determined the scaling factor for an SP34 slab phantom by performing ion measurements at various electron energies of Conv-RT according to the recommendations provided by international protocols and the percentage depth dose (PDD) to resolve some confounding points.

## Methods

### Ionometric measurement

**Fig 1:**
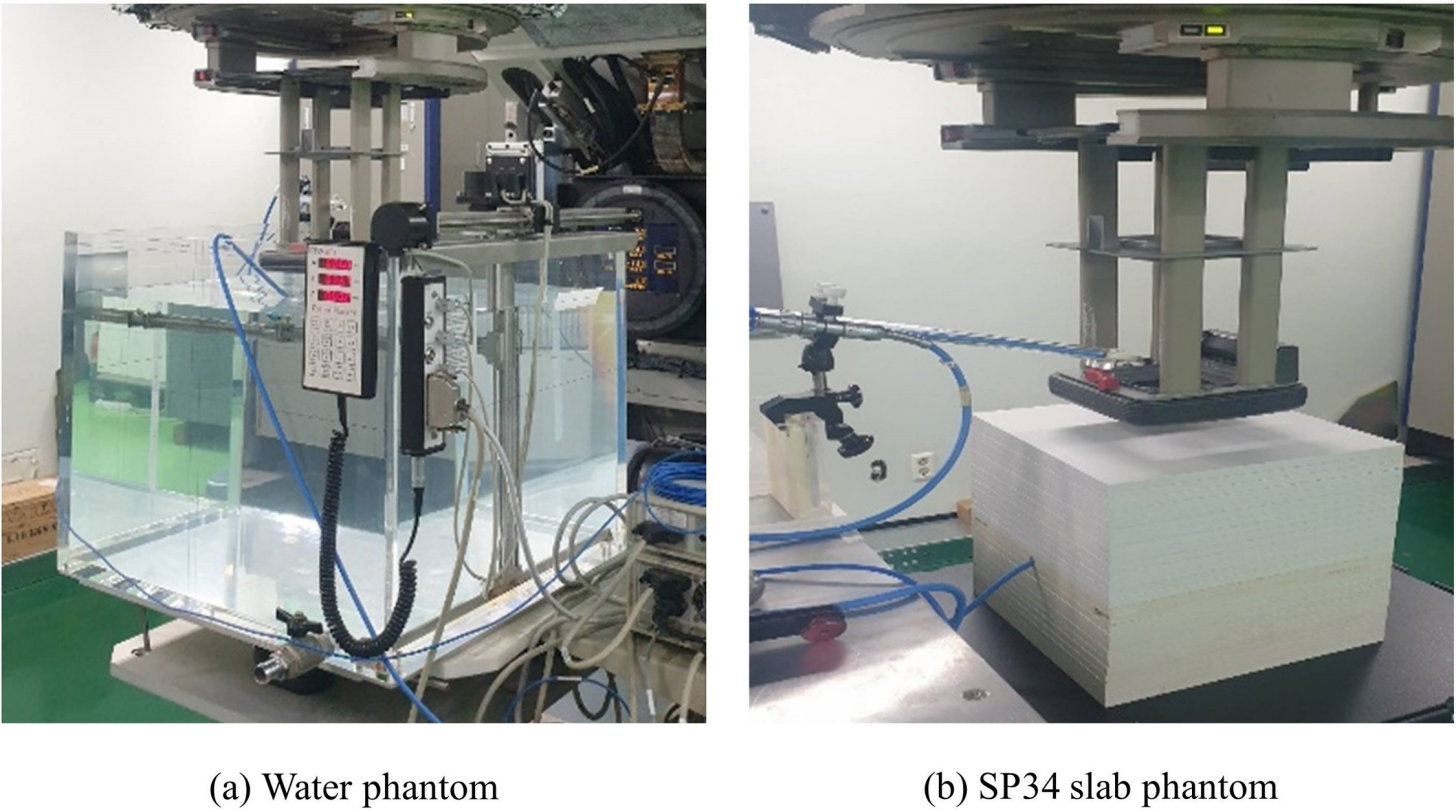
Diagram of the geometric experimental setup for percentage depth ionization. (a) Water phantom and (b) water-equivalent plastic phantom.

According to international protocol recommendations, a clinical LINAC (Varian Clinic® iX, Varian Medical System Inc., USA) was used to evaluate the depth scaling factor (denoted as c_pl_) and fluence scaling factor (denoted as h_pl_) at various electron beam energies—6, 9, 12, 16, and 20 MeV. Generally, for plastic phantoms, the behavior of the high-energy electron beam may differ from that of water, which is the reference medium, due to differences in several parameters (i.e., plastic density [denoted as ρ_pl_], electron density [denoted as ρ(r)], and effective atomic density [denoted as Z_eff_]). Therefore, to convert the measured results to those of water, the scale factor must be determined using the ionometric measurement method [15]. In the water and SP34 slab phantoms, percentage depth ionization (PDI) curves were measured. Fig 1 shows a diagram of the experiment for measuring PDI. Table 1 shows condition variables for measuring the PDI curve.

**Table 1:** Measurement conditions.

Energy	6, 9, 12, 16, and 20 MeV
**Radiation intensity**	100 MU
**Dose rate**	400 MU/min
**Source-to-surface distance**	100 cm
**Radiation field size**	10 × 10 cm

As a field chamber for PDI measurement in water, an Advanced Markus chamber (TM34045, PTW, Germany) was selected. The International Atomic Energy Agency (IAEA) TRS-398 recommends using a plane-parallel chamber to reduce scattering perturbation in electron beams with R_50_ < 4 g/cm^2^ (i.e., 10 MeV), and Baghani et al. recommended an Advanced Markus chamber with a small sensitive volume (i.e., 0.02 cm^3^) because the ion recombination correction procedure based on depth change can be ignored [17].

The electron beam quality index at 50% on the PDD curve is defined as R_50_. The Advanced Markus chamber was positioned inside the motorized 3D water phantom system (MP3 phantom, PTW, Germany) with the reference point (1.3 mm below the surface of the protection cap) on the central axis. The protection cap is 0.87-mm PMMA, which is equivalent to 1.06-mm water thickness. To compensate for the influence of clinical LINAC output fluctuations during PDI measurement, we placed a semi-flex ionization chamber (TM31010, PTW, Germany) at the edge of the irradiation surface as a reference chamber. Then, the Advanced Markus chamber was moved from the surface to 20 mm below the practical range (denoted as R_p_) using a TBA controller, and the PDI was measured toward the surface in 1-mm increments to reduce the effect of meniscus formation. We measured each ionization chamber using a dual-channel electrometer (TANDEM, PTW, Germany) and analyzed the data using commercial software (MEPHYSTO mcc Ver. 3.0, PTW, Germany).

We placed the Advanced Markus chamber inside the dedicated adoption plate and measured the PDI by manually changing the measurement point in 1-mm increments while fixing the SSD to 100 cm; because no protective cap is used for measurements in air, the reference point is located in the center of the entrance foil made of 0.03-mm polyethylene. To measure using the Advanced Markus chamber, it was connected to an electrometer (UNIDOS webline, PTW, Germany), and the reference chamber placed on the edge of the irradiation surface was connected to an independent UNIDOS webline to check the output variation of clinical LINAC. A 100-mm SP34 slab phantom was also placed at the bottom of the dedicated adoption plate to eliminate the backscattering effect in the couch. It was analyzed using free numerical analysis software (GNU Octave Ver. 5.2.0, Free Software Foundation Inc., USA). To more quantitatively analyze the PDI curve, the depth of the SP34 slab phantom into density thickness was converted, and the phantom was fitted in units of 0.01 g/cm^2^ using piecewise cubic hermite interpolating polynomial (PCHIP) interpolation; when the measurement data are insufficient, we adopted the PCHIP interpolation method, which can accurately provide the flat area without the distortion phenomenon caused by overshoot. Density thickness is determined by multiplying physical thickness by ρ_pl_.

In this study, as one of the commercially available solid-plate phantoms, we used a plastic phantom (SP34 slab phantom, IBA dosimetry, Germany) made of polystyrene suitable for quality-assurance dosimetry of photon and electron beams. The plate phantoms consist of 1 plate of 1-mm thickness, 2 plates of 2-mm thickness, 1 plate of 5-mm thickness, and 29 plates of 10-mm thickness; according to the manufacturer’s recommendations, the external dimensions are designed such that a suitable combination of slab phantoms in a 300-mm regular hexahedron can measure up to 250 mm in 1-mm increments for the energy range of 0.1–50 MeV for photon beams and 2–50 MeV for electron beams. Furthermore, it contains polystyrene C8H8 (type RW3 materials, composition: 98% polystyrene + 2% TiO_2_), which has ρ_pl_ of 1.045 g/cm^3^, Z_eff_ of 5.74, and ρ(r) of 1.01 [18–20].

In this study, the mass was divided by the volume to calculate the density to experimentally determine the ρ_pl_ of the SP34 slab phantom. A precision digital balance (FX-3000i, A&D Co., Japan) was used to determine the mass, and a stainless straight rule (1,000-mm measure range and 0.5-mm resolution, HARA, Japan) was used to determine the volume. Then, a vernier caliper (150-mm measure range and 0.02-mm resolution, MITUTOYO Co., Japan) was used to measure physical thickness.

### Evaluation of scaling factor

The PDI curve and c_pl_ were derived using ionometric measurements to use the SP34 slab phantom in a nonreference condition. We summarized the relationship between physical quantities and scaling factors as follows: it is derived from Equation (7.10) in the international protocol IAEA TRS-398 recommendations [15].

$$c_{pl}=R_{50,ion}\cdot R_{50,ion,pl}{}^{-1}\left(R_{50,ion,pl}\;in\;g\cdot cm^{-2}\right)$$ (1)

where R_50,ion_ represents half of the PDI in water and R_50,ion,pl_ represents half of the PDI in the plastic phantom. The density thickness of each material should be used to calculate R_50,ion_ and R_50,ion,pl_. Using the c_pl_ calculated using Eq (2), the depth on the density thickness side can be converted to a water-equivalent depth in the SP34 slab phantom; it is derived from Equation (7.9) in the international protocol IAEA TRS-398 recommendations [15].

$$z_{w}\left[g\cdot cm^{-2}\right]=z_{pl}\cdot c_{pl}\left(z_{pl}\;in\;g\cdot cm^{-2}\right)$$ (2)

where z_w_ represents water-equivalent depth and z_pl_ represents depth in terms of density thickness in plastic phantoms. Because of the difference in electron fluence spectra, the reading value measured using the ionization chamber is not the same as the reading value measured in water, the actual reference medium, when converted to z_w_. This is one of the factors that limit the clinical use of various plastic phantoms. To correct this, the international protocol IAEA TRS-398 recommends using h_pl_, which can be calculated using the following formula; it is derived from Equation (7.12) in the international protocol IAEA TRS-398 recommendations [15].

$$h_{pl}=M_{Q}\cdot M_{Q,pl}{}^{-1}$$ (3)

where M_Q_ is the chamber reading at the reference depth in water (denoted as z_ref_), M_Q,pl_ is the chamber reading at the reference depth in the plastic phantom (denoted as z_ref,pl_). The calculation formula for the reference depth for measurement inside the phantom differs depending on the material of the phantom. The reference depth in the water phantom is defined by the following formula; it is derived from Equation (7.2) in the international protocol IAEA TRS-398 recommendations [15].


$$z_{xef}\left[g/cm^{2}\right]=0.6\cdot R_{50}-0.1\left(R_{50}\;in\;g\cdot cm^{-2}\right)$$ (4)

The measured R_50,ion_ was used to determine z_ref_. At this time, the relationship between R_50_ and R_50,ion_ is defined by the following formula; it is derived from Equation (7.1) in the international protocol IAEA TRS-398 recommendations [15].


$$\begin{array}{c} R_{50}=1.029\cdot R_{50,ion}-0.06\left(R_{50,ion}\leq 10g\cdot cm^{-2}\right)\\ R_{50}=1.059\cdot R_{50,ion}-0.37\left(R_{50,ion}>10g\cdot cm^{-2}\right) \end{array}$$ (5)

We placed the reference point of the Advanced Markus chamber inside the phantom so that it was located on the central axis and moved it to z_ref_ using a TBA controller unit to measure M_Q_ in water. The electrometer was measured using UNIDOS webline. However, the chamber must be positioned at the scaled reference depth z_ref,pl_ in the plastic to determine the absorbed dose to water at z_ref_ in water using a plastic phantom. z_ref,pl_ is defined by the following formula; it is derived from Equation (7.11) in the international protocol IAEA TRS-398 recommendations [15].

$$z_{ref,pl}\left[g\cdot cm^{-2}\right]=z_{ref}\cdot c_{pl}{}^{-1}\left(z_{ref}\;in\;g\cdot cm^{-2}\right)$$ (6)

h_pl_ calibration at the z_ref,pl_ position of the SP34 slab phantom can be used to check the output dose of the equipment. The PDD curve (denoted as PDD_pl,IC_) measured in the SP34 slab phantom using the Advanced Markus chamber was compared with the PDD curve (denoted as PDD_w,IC_) measured in water as a reference medium to validate the experimentally determined scaling factor; the PDD curve was derived by applying the MEPHYSTO mcc water-to-air stopping power ratio (S_w,air_) to the PDI curve. Furthermore, several parameters that represent electron beam characteristics were compared: z_max_, z_ref_, R_90_, and R_50_. z_max_ is the maximum dose depth and R_90_ is the clinical therapeutic dose range.

### Radiochromic film calibrations

**Fig 2:**
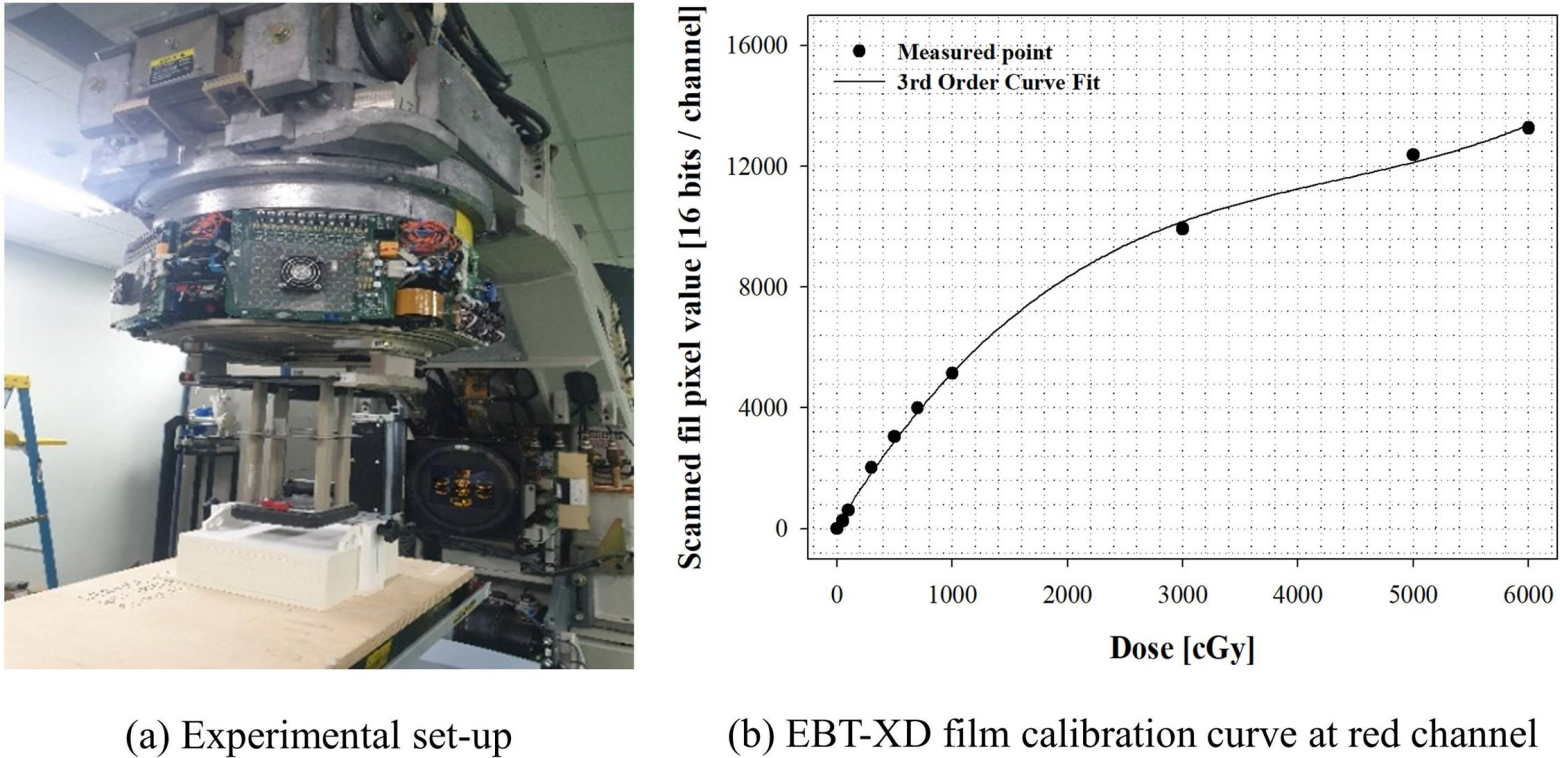
Experimental setup for radiochromic film calibration.

In this study, the radiation-sensitive film EBT-XD (Ashland Inc., Covington, KY), which is widely used for dosimetry under nonreference conditions, was selected. When calibrated through the red channel at 6 and 20 MeV, the uncertainty in the range of 5–40 Gy is within 2.3% in the case of EBT-XD film [21]. We used a 9-MeV electron beam to obtain a calibration curve for the EBT-XD film; the manufacturer’s note presents the energy dependence for the EBT-XD film as “<5% difference in net optical density when exposed at 100 keV and 18 MeV.” The EBT-XD film was placed in the SP34 slab phantom at the maximum dose depth and irradiated in 10 steps in the range of 0–60 Gy at an SSD of 100 cm and field size of 10 × 10 cm; the manufacturer’s memo recommends a dynamic dose range of 0.1–60 Gy and a dose optimum range of 0.4–40 Gy. Fig 2 shows the experimental state and the calibration curve used to secure the calibration curve for the EBT-XD film. After irradiation, it was scanned at a resolution of 48-bit RGB (16 bits per channel) and 72 dots per inch (DPI) using a flatbed scanner (Epson Expression XL10000, EPSON America, Inc., USA). Then, the red channel calibration curve was obtained using commercially available software (DoseLab Ver. 6.80, Mobius Medical Systems, USA). The unit dose analysis values for 6 and 20 MeV have been reported to vary within 0.5% of 10 Gy and <3% of 10 Gy [21].

### Evaluation of PDD using EBT-XD

In this study, to evaluate the possibility of film dosimetry using the SP34 slab phantom and EBT-XD, PDD was used. We irradiated 4,000 MU at 400 MU/min to include the optimum dose range (0.4–40 Gy) of the EBT-XD film in the overall PDD curve. Table 2 shows condition variables for measuring the film dosimetry.

**Table 2:** Measurement conditions.

**Energy**	6, 9, 12, 16, and 20 MeV
**Radiation intensity**	4,000 MU
**Dose rate**	400 MU/min
**Source-to-surface distance**	100 cm
**Radiation field size**	10 × 10 cm

**Fig 3:**
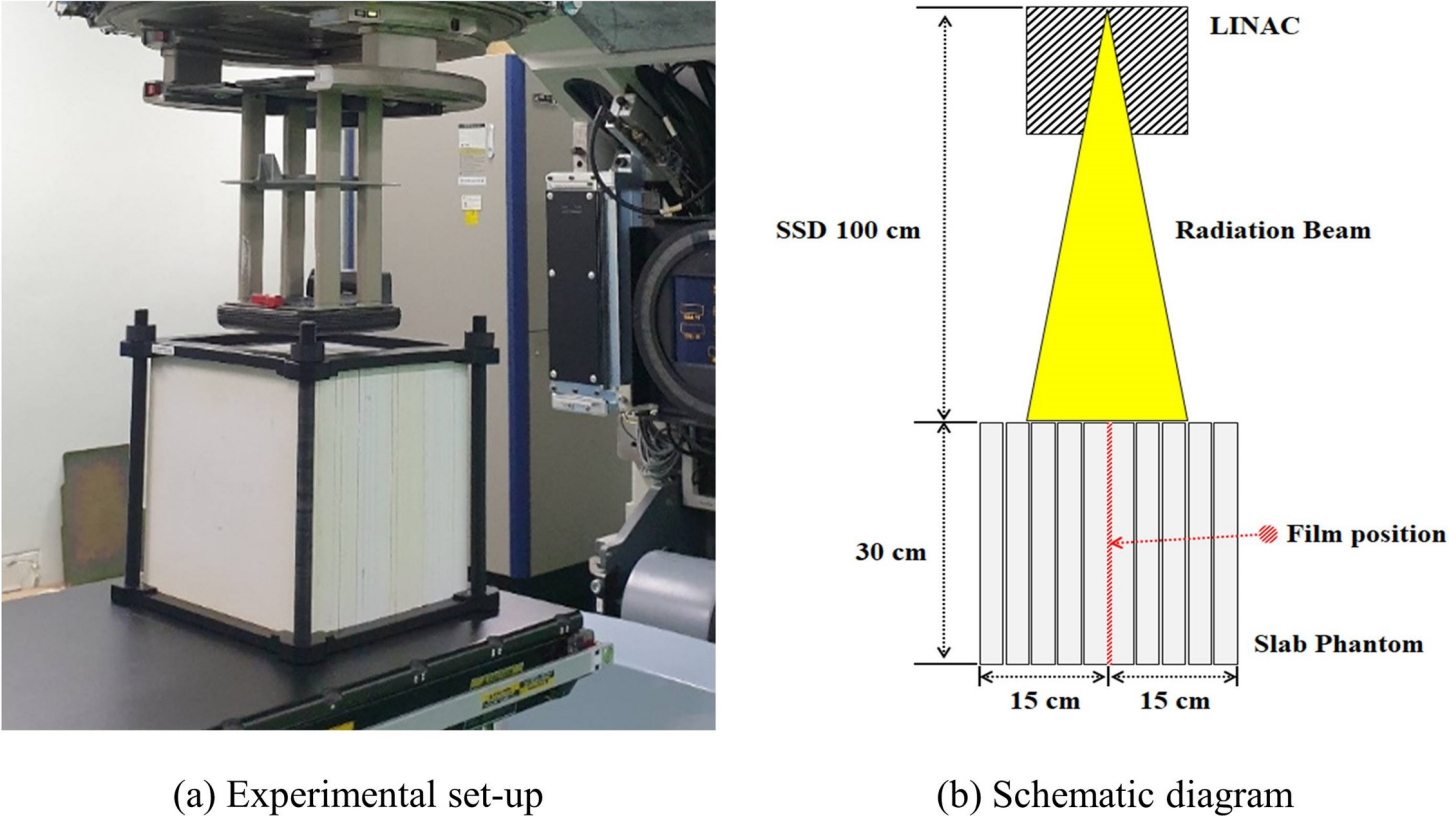
Diagram of the geometric experimental setup for percentage depth dose.

In the SP34 slab phantom, to evaluate the PDD curve, we placed an EBT-XD film with dimensions of 20.3 × 25.4 cm parallel to the central axis. For lateral electronic equilibrium, an EBT-XD film was inserted in the middle of a 30-cm SP34 slab phantom. Fig 3 shows a diagram of an experiment for evaluating PDD curves using an EBT-XD film. After 24 h of irradiation, image data were collected under the same conditions as when the calibration curve was obtained using an Epson Expression XL10000 scanner, and DoseLab was used to analyze the dose distribution for the red channel. The depth profile for the central axis was analyzed and converted to physical thickness using pixel (i.e., 72 DPI = 0.0352778 cm) information.

Several assumptions were compared to clearly understand the statement that correction of the measured depth dose proposed in the American Association of Physicists in Medicine (AAPM) TG-25 protocol is not required: (i) PDD_pl_,_RCFs,w/o_: the PDD curve assuming water-equivalent thickness by the physical thickness; (ii) PDD_pl,RCFs,ρ_: the PDD curve assuming water-equivalent thickness by the density thickness; (iii) PDD_pl,RCFs,c_: the PDD curve assuming water-equivalent thickness by the density thickness applied with c_pl_. We used PCHIP interpolation to fit various PDD curves in units of 0.01 g/cm^2^ to quantitatively analyze them. In this study, various PDD curves analyzed according to the application of correction factors (i.e., ρ_pl_, c_pl_) in film dosimetry were compared with PDD_w,IC_; several parameters (i.e., z_max_, R_90_, and R_50_) representing electron beam characteristics were analyzed.

## Results and discussion

### Evaluation of PDI

**Fig 4:**
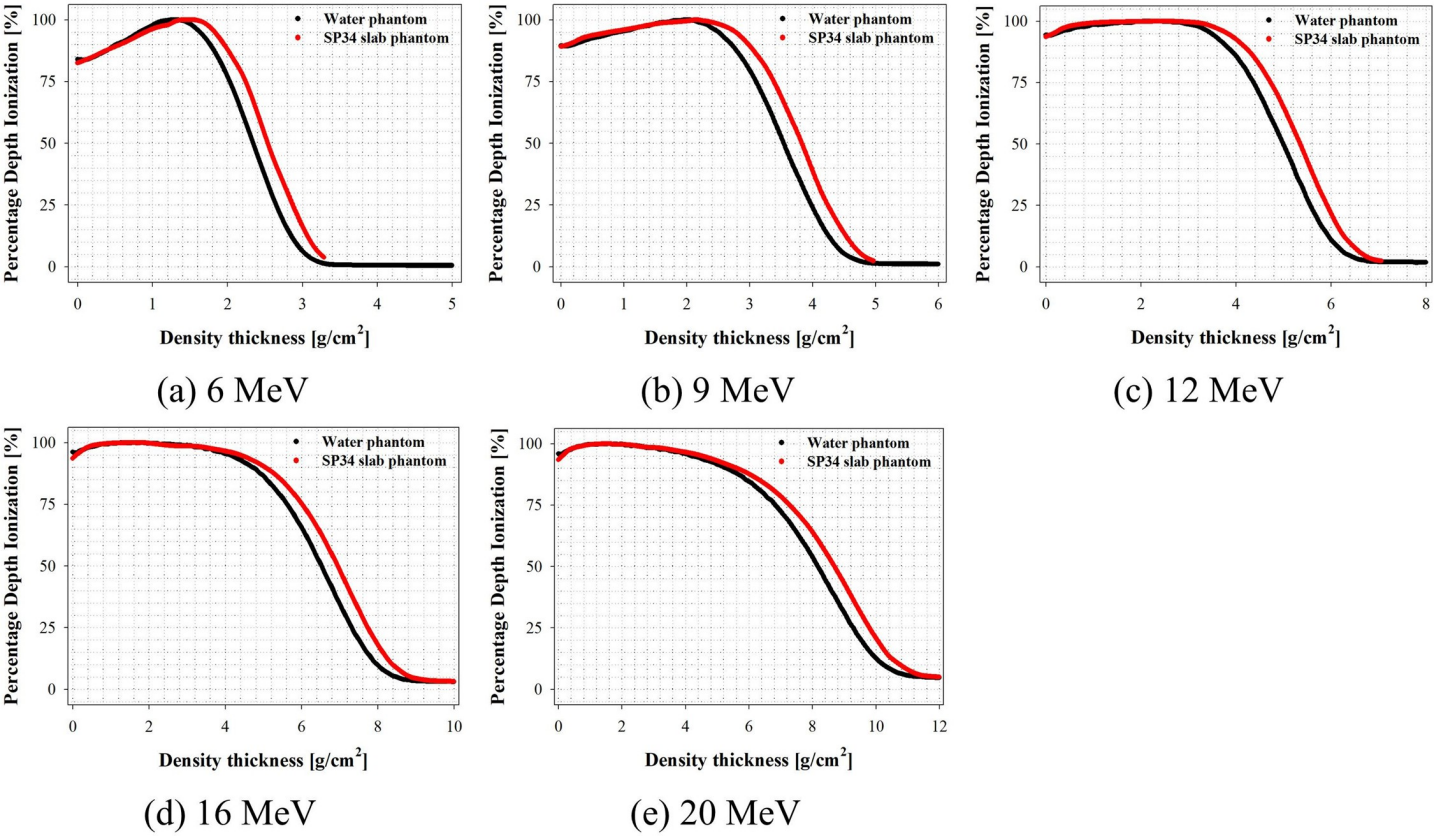
Percentage depth ionization as a function of density thickness. The plastic density (ρ_pl_) is calibrated to the physical thickness of the SP34 slab phantom to determine the density thickness.

In this study, to quantitatively evaluate the scaling factor in electron beams of various energies according to international protocol recommendations, PDI curves were measured in water as a reference medium and a SP34 slab phantom using an Advanced Markus chamber using clinical LINAC. Fig 4 shows the PDI curve results as a function of electron beam energy.

The PDI curve measurement confirmed bremsstrahlung contamination at all electron beam energies in the case of water as a reference medium but not below 12 MeV in the case of the SP34 slab phantom. As illustrated in Fig 1, measuring depths >6 MeV and 3.3 g/cm^2^, 9 MeV and 5.0 g/cm^2^, and 12 MeV and 7.1 g/cm^2^ were impossible.

Furthermore, the PDI curve measured through the SP34 slab phantom was biased to the right at all energies compared with the results measured in water, the reference medium. This means that the electron beam’s behavior mechanism (i.e., elastic and inelastic collision and scattering) differs between media due to differences in several parameters (i.e., ρ_pl_, Z_eff_, and ρ(r)). These findings are supported by the mass stopping power difference based on the electron beam energy presented by the National Institute of Standards and Technology. It is a physical quantity representing the rate of energy loss per density thickness of the medium. Polystyrene (ρ_pl_ of 1.06 g/cm^3^) (6 MeV and 3.6% and 20 MeV and 5.5% lower, respectively, than water) resulted in different energy and electron distributions at the same depth [22]. Therefore, the scaling factor is important for converting the ionometric measurement result of the SP34 slab phantom to the result measured in water.

### Evaluation of depth scaling factor (c_pl_)

**Fig 5:**
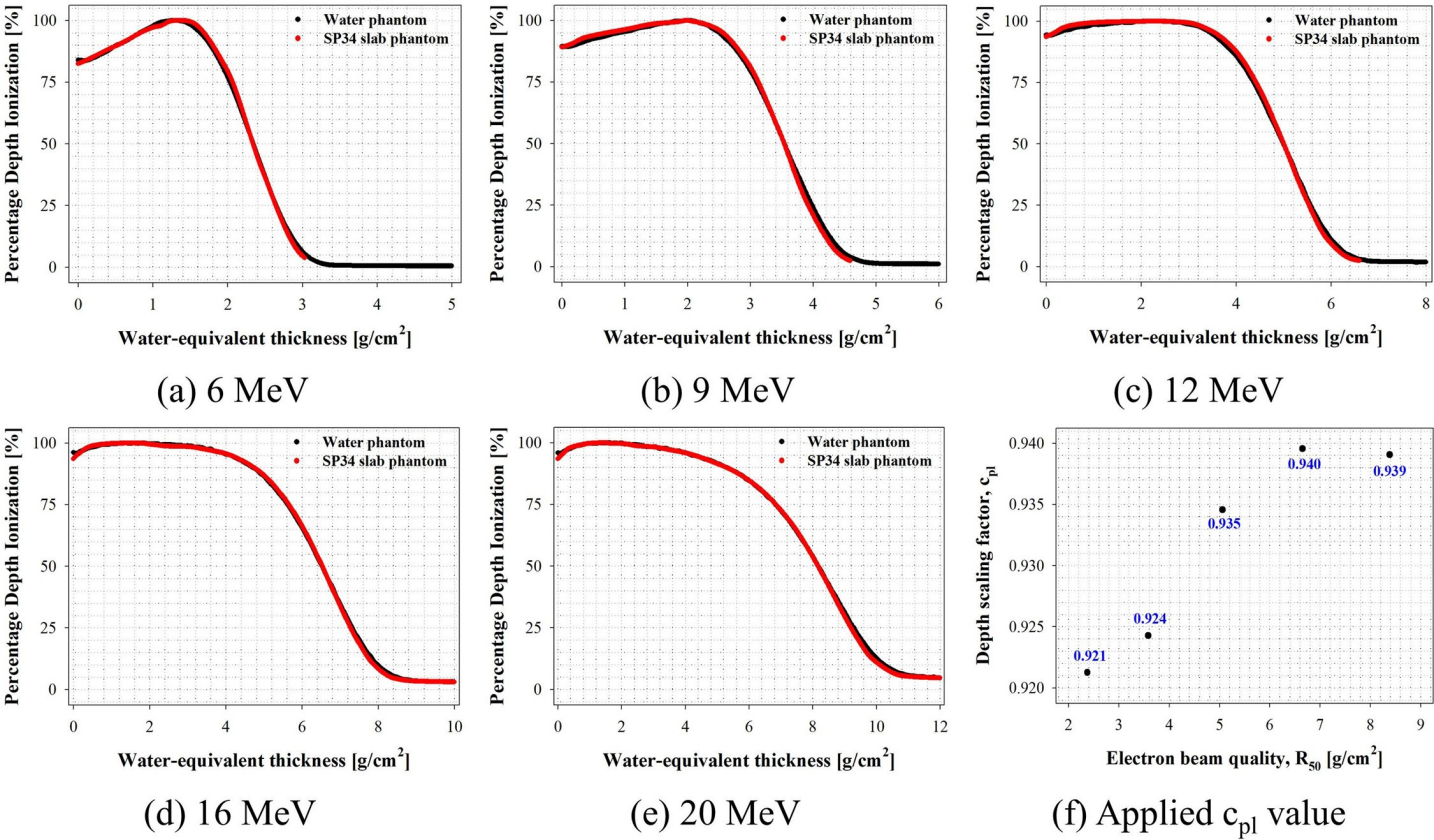
Percentage depth ionization as a function of water-equivalent thickness. The depth scaling factor (c_pl_) is calibrated to the density thickness of the SP34 slab phantom to determine the water-equivalent thickness.

In this study, c_pl_ was derived from the PDI curve obtained using the SP34 slab phantom in dosimetry. Eq (1) shows that the physical quantities associated with the depth scaling factor are “R_50,ion_” and “R_50,ion,pl_.” Table 3 shows the parameters for calculating the depth scaling factor. The c_pl_ value increased as the electron beam energy increased at R_50_ ≤ 6.65 g/cm^2^ (i.e., 16 MeV); however, the table showed a decrease at higher values. The cubic function “Y = −0.00068 X^3^ + 0.00915 X^2^ − 0.03365 X + 0.95873” follows R^2^ = 1.00000. By averaging the calculated c_pl_ values for the SP34 slab phantom at electron beam energies of R_50_ <4.0 g/cm^2^ (i.e., 10 MeV), we obtained 0.923. The international protocol IAEA TRS-398 specifies the c_pl_ value of white polystyrene as 0.922, which is within 0.11% of the result of this study. The PDI curve applied in the experimentally determined c_pl_ is shown in Fig 5.

**Table 3:** Parameters related to c_pl_ in PDI curves measured at various electron energies.

Parameter (g/cm^2^)	Energy (MeV)				
	6	9	12	16	20
R_50,ion_	2.34	3.54	5.00	6.53	8.17
R_50,ion,pl_	2.54	3.83	5.35	6.95	8.70
c_pl_	0.921	0.924	0.935	0.940	0.939

### Evaluation of fluence scaling factor (h_pl_)

**Fig 6:**
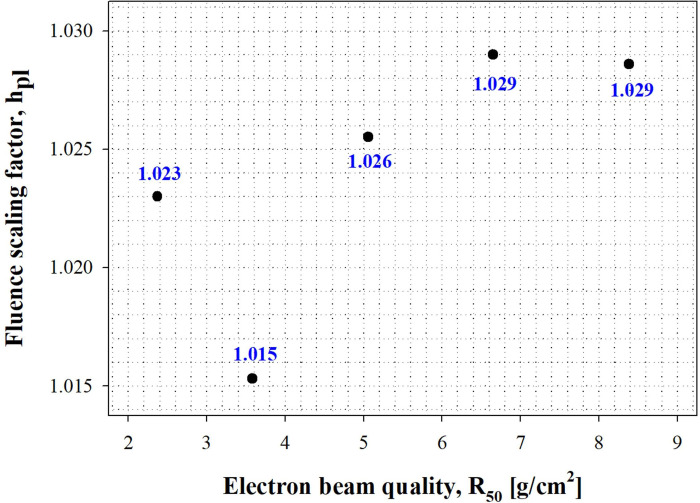
Calculated fluence scaling factor from ionometric measurement at the reference depth.

In this study, to correct differences in reading values between each medium, h_pl_ was derived from the reference depth of each medium. As shown in Eq (3), two physical quantities were found to be related to the fluence scaling factor: “M_Q_” and “M_Q,pl_.” The ionometric measurement depths for determining h_pl_ in the water and SP34 slab phantoms are shown in Table 4. The measurement depth is given in terms of the density thickness of each medium. Fig 6 shows the h_pl_ as the electron beam quality changes.

**Table 4:** Measurement depth for determining the fluence scaling factor.

Parameter (g/cm^2^)	Energy (MeV)				
	6	9	12	16	20
z_ref_^a^	1.32 (1.30)	2.05 (2.00)	2.94 (3.00)	3.89 (3.90)	4.93 (4.90)
z_ref,pl_^b^	1.43 (1.46)	2.22 (2.19)	3.14 (3.14)	4.14 (4.18)	5.25 (5.23)

^a^ The z_ref_ calculated using Eq (4) is presented, and the actual measured depth in the water phantom is shown in brackets; according to the notation of significant figures of the z_ref_ value provided in the IAEA TRS-398 worksheet, an error of <0.5 mm occurs.

^b^ The z_ref,pl_ calculated using Eq (6) is presented, and the actual measured depth in the SP34 slab phantom is shown in brackets; because the minimum thickness of the SP34 slab phantom is 1 mm, an error within a density thickness of 0.05 g/cm^2^ occurs.

The h_pl_ value decreased as the electron beam energy increased at R_50_ ≤ 3.58 g/cm^2^ (i.e., 9 MeV) but increased at 3.58 g/cm^2^ < R_50_ ≤ 6.65 g/cm^2^ (i.e., 9–16 MeV), and no change above that was observed. Averaging the calculated h_pl_ values for the SP34 slab phantom at electron beam energies within R_50_ < 4.0 g/cm^2^ (i.e., 10 MeV) yielded 1.019. The international protocol IAEA TRS-398 specifies the h_pl_ value of white polystyrene as 1.019, which is within 0.01% of the result of this study.

Based on these findings, when electron dosimetry of an electron beam within R_50_ ≤ 4.0 g/cm^2^ (i.e., 10 MeV) is performed in clinical practice with an SP34 slab phantom, the white polystyrene provided in the IAEA TRS-398 worksheet is used. However, because it cannot reflect changes in the scaling factor due to electron beam energy changes, it should only be used for routine quality assurance.

### Evaluation of PDD

**Fig 7:**
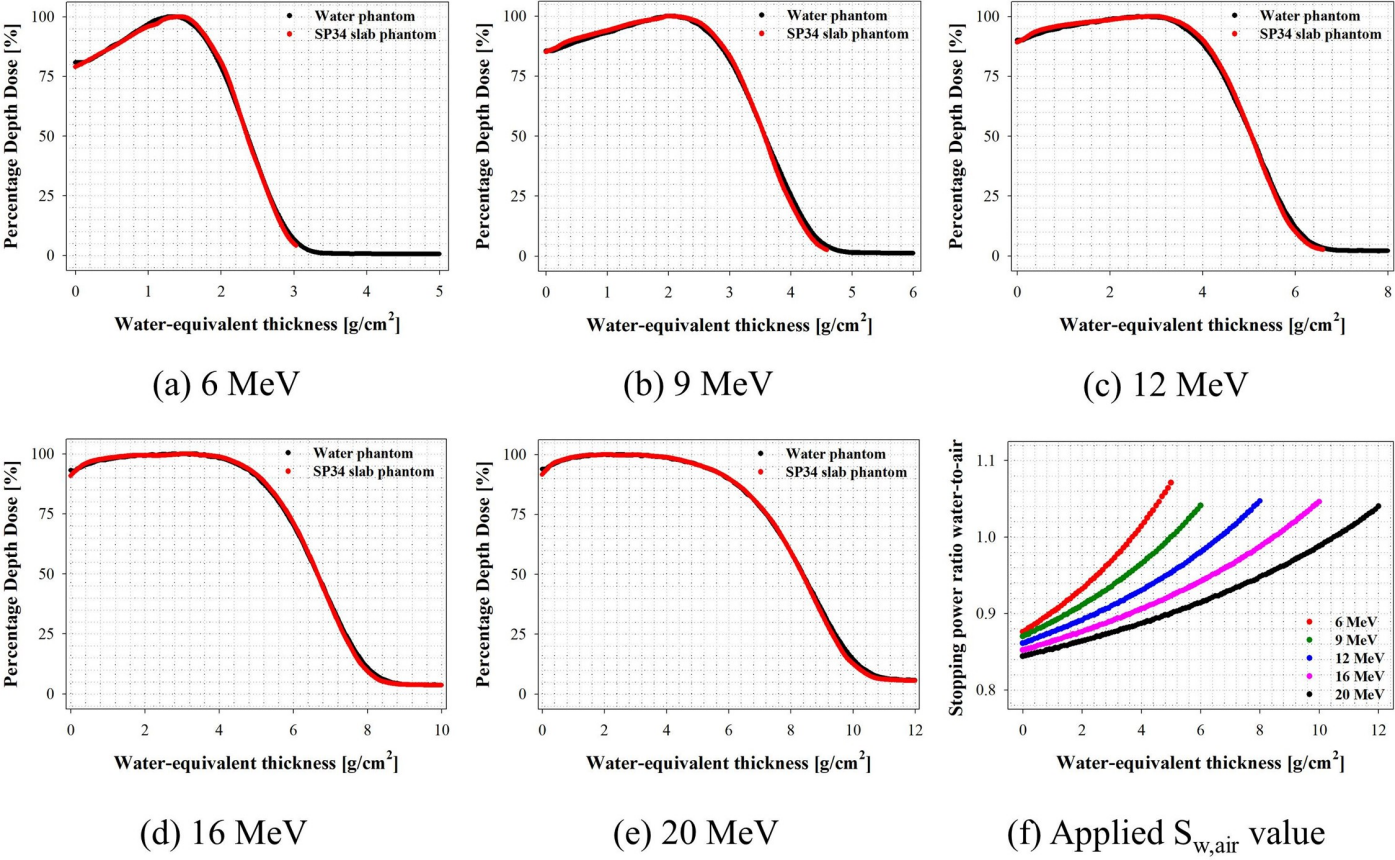
Percentage depth dose as a function of water-equivalent thickness using the ionization chamber.

An Advanced Markus chamber was used to calculate PDD curves for each medium to validate the scaling factor determined experimentally in electron beams of various energies using clinical LINAC. Fig 7 shows the PDD curve results as a function of electron beam energy. Table 5 shows the parameters associated with the electron beam characteristics in the measured PDD curve.

**Table 5:** Parameters associated with electron beam characteristics in PDD curves measured at various electron energies.

Energy (MeV)		Parameters							
		Range (g/cm^2^)				PDD (%)			E_0_^c^ (MeV)
		z_max_	z_ref_^a^	R_90_	R_50_	z_s_^b^	z_ref_^a^	1 g/cm^2^	
6	PDD_w,IC_	1.30	1.32	1.80	2.37	80.7	100.0	96.8	5.7
	PDD_pl,IC_	1.43	1.43	1.83	2.36	79.7	100.0	95.8	5.6
9	PDD_w,IC_	2.00	2.05	2.77	3.58	85.5	100.0	93.1	8.3
	PDD_pl,IC_	2.01	2.22	2.81	3.57	85.6	99.5	93.9	8.3
12	PDD_w,IC_	2.80	2.94	3.93	5.06	90.0	99.7	95.7	11.6
	PDD_pl,IC_	2.99	3.14	4.02	5.06	89.6	99.7	96.3	11.6
16	PDD_w,IC_	3.00	3.89	5.05	6.65	92.8	98.8	97.7	15.3
	PDD_pl,IC_	3.13	4.14	5.11	6.65	91.6	98.2	98.2	15.3
20	PDD_w,IC_	2.60	4.93	5.97	8.38	93.8	96.0	98.8	19.5
	PDD_pl,IC_	2.09	5.25	5.99	8.37	92.3	94.6	98.8	19.4

^a^ z_ref_ means z_ref_ in the water phantom at PDD_w,IC_, and z_ref,pl_ means z_ref_ in the SP34 slab phantom at PDD_pl,IC_.

^b^ z_s_ is the measurement depth of skin dose and is defined as 0.05 g/cm^2^ in the AAPM TG-25 protocol [16].

^c^ E_0_ is the average energy on the surface of the phantom and can be calculated using “0.022·(R_50_)^2^ + 2.059·(R_50_) + 0.656 (cm).” [16]

The parameters were examined, and it was found that R_50_ was well matched within 0.01 g/cm^2^ in all electron beam energy ranges. Because R_50_ matches well when the scaling factor is used, it is assumed that the average energy formula of the phantom surface can be used in the SP34 slab phantom. Furthermore, R_50_ ≤ 5.06 g/cm^2^ (i.e., 12 MeV) was confirmed to be within 1% of the overall PDD curve. However, it was within 1.5% at higher electron beam energies.

### Electron film dosimetry using the SP34 slab phantom

**Fig 8:**
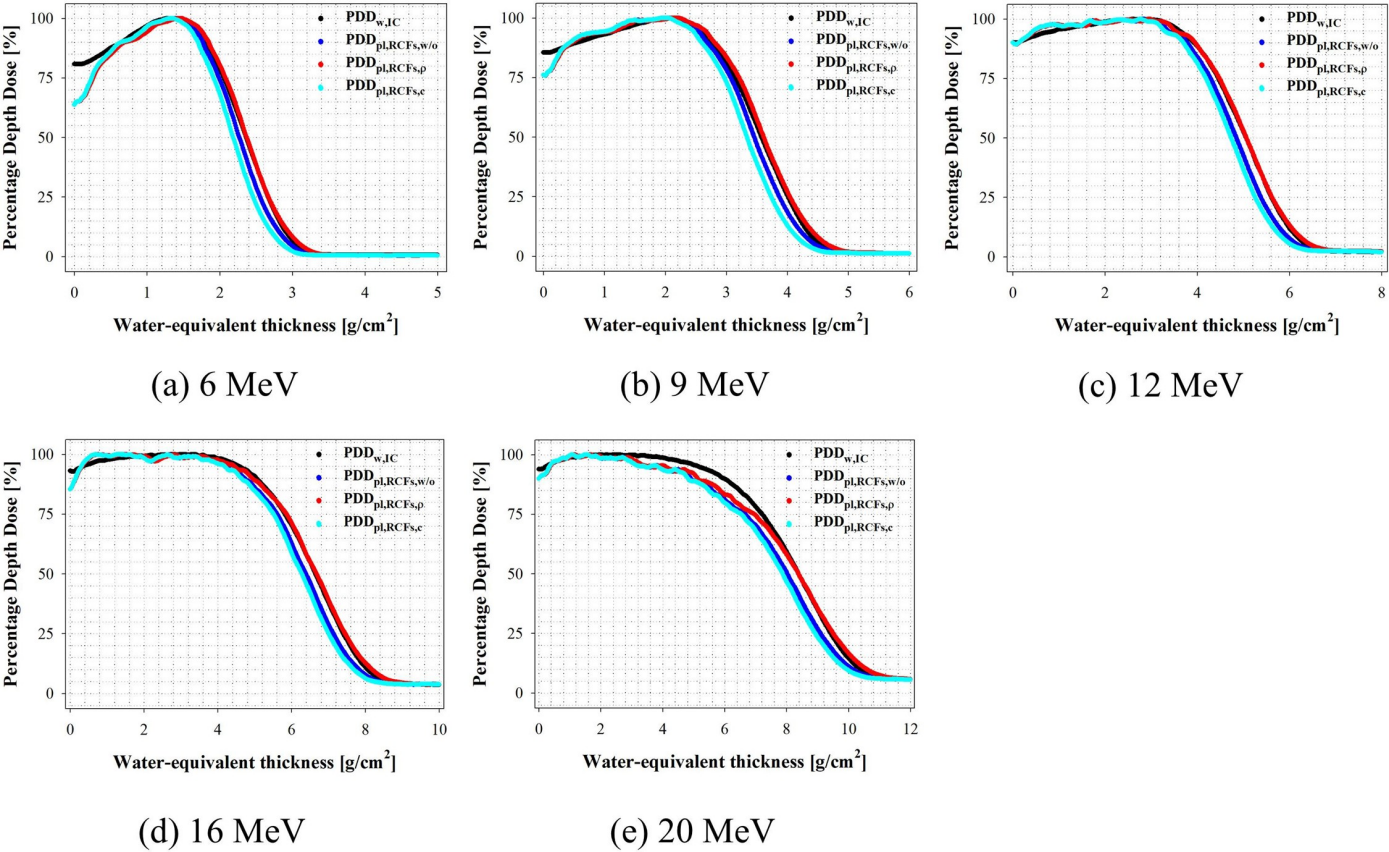
Percentage depth dose as a function of water-equivalent thickness using film dosimetry.

In this study, the validity of the application of correction factors (i.e., ρ_pl_ and c_pl_) was validated when performing electron film dosimetry with the SP34 slab phantom. Various PDD curves measured using the Advanced Markus chamber in water as a reference medium were compared with those measured using electron film dosimetry in the PDD_w,IC_ and SP34 slab phantoms. Fig 8 shows the PDD curve results as a function of electron beam energy.

The PDD curve analysis revealed an underestimation of the dose in the build-up region of the overall electron beam energy. This dose distortion is an artifact occurring when measuring PDD curves using film dosimetry and does not appear in Advanced Markus chambers. J. Dutreix and A. Dutreix reported that when performing electron film dosimetry using a plastic phantom, two types of film artifacts could occur. (a) Air gaps are found on both sides of the film, and (b) the film edge cannot be adjusted to the phantom surface [23]. Therefore, when performing electron film dosimetry, strictly adhering to film positioning and air gaps, which contribute to artifacts, is important.

We attempted to minimize the air gap in the SP34 slab phantom using a dedicated jig when performing electron film dosimetry; however, due to the air gaps caused by the physical thickness of the EBT-XD film (i.e., 0.275 mm), type (a) artifacts occurred. It is assumed that type (a) artifacts are a fundamental problem that cannot be solved in an air environment using a plastic phantom, and their effects are more pronounced at R_50_ ≥ 5.06 g/cm^2^ (i.e., 12 MeV). Additionally, to prevent type (a) film artifacts, we adjusted the EBT-XD film to match the surface of the SP34 slab phantom as much as possible and fixed it with tape. El. Barouky and Jad proposed placing an ultrasound transmission gel and two other RCFs on the surface to avoid dose underestimation in the build-up area [24]. However, because our purpose was to validate the application of the correction factor when performing electron film dosimetry, we did not solve the dose distortion in the build-up area. The PDD curve analysis in this study revealed that PDD_pl,RCFs,ρ_ and PDD_w,IC_ had the most similar trend. Table 6 shows the parameters that were found to be related to the electron beam characteristics in the PDD curve.

**Table 6:** Parameters related to electron beam characteristics in the PDD curves measured at various electron energies.

Energy (MeV)		Parameters					
		Range (g/cm^2^)			PDD (%)		E_0_ (MeV)
		z_max_	R_90_	R_50_	z_s_	1 g/cm^2^	
6	PDD_w,IC_	1.31	1.80	2.37	80.7	96.8	5.7
	PDD_pl,RCFs,w/o_	1.38	1.77	2.27	65.1	95.4	5.4
	PDD_pl,RCFs,ρ_	1.45	1.85	2.37	65.1	94.3	5.7
	PDD_pl,RCFs,c_	1.33	1.70	2.19	65.1	96.5	5.3
9	PDD_w,IC_	2.00	2.77	3.58	85.5	93.1	8.3
	PDD_pl,RCFs,w/o_	2.08	2.67	3.46	76.3	94.2	8.0
	PDD_pl,RCFs,ρ_	2.18	2.79	3.61	76.1	94.1	8.4
	PDD_pl,RCFs,c_	2.01	2.58	3.34	76.3	94.3	7.8
12	PDD_w,IC_	2.80	3.93	5.06	90.0	95.7	11.6
	PDD_pl,RCFs,w/o_	2.82	3.79	4.85	89.4	97.3	11.2
	PDD_pl,RCFs,ρ_	2.95	3.96	5.07	89.4	97.6	11.7
	PDD_pl,RCFs,c_	2.76	3.70	4.74	89.4	97.2	10.9
16	PDD_w,IC_	3.05	5.05	6.65	92.8	97.7	15.3
	PDD_pl,RCFs,w/o_	1.31	4.72	6.39	86.4	99.4	14.7
	PDD_pl,RCFs,ρ_	1.36	4.93	6.68	86.3	99.4	15.4
	PDD_pl,RCFs,c_	1.28	4.64	6.28	86.4	99.4	14.5
20	PDD_w,IC_	2.55	5.97	8.38	93.8	98.8	19.5
	PDD_pl,RCFs,w/o_	1.09	4.91	8.03	90.7	99.4	18.6
	PDD_pl,RCFs,ρ_	1.14	5.13	8.39	90.6	99.0	19.5
	PDD_pl,RCFs,c_	1.07	4.81	7.88	90.7	99.5	18.2

## Conclusion

In this study, we derived the results for the SP34 slab phantom at various electron energies of Conv-RT with clearly known beam characteristics to verify and address confusing points about electron film dosimetry using white polystyrene, which is briefly presented in international protocols. To determine the scaling factor experimentally, we performed ionometric measurements on water as a reference medium and an SP34 slab phantom using an Advanced Markus chamber and compared it with white polystyrene, as recommended by international protocols. White polystyrene has a ρ_pl_ of 1.06 g/cm^3^; however, an SP34 slab phantom has a ρ_pl_ of 1.045 g/cm^3^, which causes confusion because the physical properties are different. Additionally, whether correction factors are used when performing electron film dosimetry will be discussed. Scaling factor analysis using ionometric measurements yielded c_pl_ of 0.923 and h_pl_ of 1.019 for the SP34 slab phantom at an electron beam energy of R_50_ <4.0 g/cm^2^ (i.e., 10 MeV). This had a value similar to that of white polystyrene (i.e., c_pl_ of 0.922 and h_pl_ of 1.019). Based on these findings, it was determined that white polystyrene and SP34 slab phantoms have properties comparable with those of water-equivalent plastic phantoms.

PDD curve analysis on the presence or absence of correction factors in electron film dosimetry revealed that PDD_pl,RCFs,ρ_ was measured in the standard medium using an Advanced Markus chamber, assuming the density thickness calculated by applying ρ_pl_ to the physical thickness of the SP34 slab phantom as the water-equivalent thickness. It was found to be comparable with the PDD_w,IC_. The recommendation in the international protocol that no correction for the measured depth dose is required implies that no scaling factor correction is required for the SP34 slab phantom. That is, the density thickness of a water-equivalent plastic phantom can be assumed to be a water-equivalent thickness. This may make film dosimetry more useful in determining beam characteristics during the clinical LINAC development stage.

In this study, we confirmed two potential misunderstandings while determining beam characteristics using electron film dosimetry. In the future, we hope to be able to use it as basic data for clinical LINAC research, such as electron FLASH-RT and intraoperative electron radiotherapy.
